# The impact of the number of lymph nodes removed with D2 lymph node dissection on survival in gastric cancer

**DOI:** 10.1590/1806-9282.20250506

**Published:** 2025-10-17

**Authors:** Orhan Üreyen, Emrehan İnci, Hatice Şimşek, Merve Hamzaçelebioğlu Şahin, Asuman Argon, Enver İlhan

**Affiliations:** 1 University of Health Sciences Turkey, İzmir City Hospital, İzmir Faculty of Medicine, Department of General Surgery – İzmir, Türkiye.; 2 Dokuz Eylul University, Faculty of Medicine, Department of Public Health – İzmir, Türkiye.; 3 University of Health Sciences Turkey, İzmir City Hospital, İzmir Faculty of Medicine, Department of Pathology Clinic – İzmir, Türkiye.

**Keywords:** Gastric cancer, Surgery, Lymph node excision, Survival

## Abstract

**OBJECTIVE:**

The relationship between the number of lymph nodes removed and survival was investigated in patients undergoing radical gastrectomy and D2 lymph node dissection for gastric cancer.

**METHODS:**

Patients who underwent gastrectomy and D2 lymph node dissection were analyzed. The cases were evaluated based on age, sex, tumor stage, number of removed lymph nodes, operation duration, presence of comorbidities, tumor status at the surgical margin, lymphovascular invasion, and perineural invasion. Survival analyses at 1 and 3 years were conducted.

**RESULTS:**

The 1-year survival rate was 73%, and the 3-year survival rate was 40.4%. Factors associated with 1-year survival included age ≥70 years, the number of removed lymph nodes <35, and the presence of lymphovascular invasion, which negatively impacted survival. Age ≥70 years reduced survival by 3.7 times, the number of lymph nodes removed <35 reduced survival by 3.3 times, and the presence of lymphovascular invasion reduced survival by 4.6 times. For 3-year survival, age affected survival by 4.1 times, and tumor presence at the surgical margin affected survival by 2.85 times. A positive correlation was found between the number of removed lymph nodes and survival (p=0.006).

**CONCLUSION:**

Lymph node dissection should be performed according to guidelines, and efforts should be made to increase the number of removed lymph nodes.

## INTRODUCTION

According to GLOBOCAN 2020 data, gastric cancer ranks 5th in the world in terms of frequency and 4th in cancer-related deaths^1^. The cornerstone of treatment for long-term survival is radical gastrectomy and lymph node (LN) dissection^2^. The optimal extent of lymphadenectomy has been a long-standing debate^3^. D1 and D2 dissections have been compared, and it has been demonstrated that D2 dissection provides survival advantages. Currently, D2 or D2+LN dissection is the standard procedure of lymphadenectomy for gastric cancer. Compared to D1, D2 LN dissection is an extended LN dissection aimed at prolonging patient survival and clearing potential metastatic LNs^4^. Most guidelines^5,6^ advocate performing D2 lymphadenectomy in specialized centers with experienced surgeons, though that is not always applied as standard therapy outside East Asia. LN status is a significant prognostic factor and plays a crucial role in postoperative treatment decisions for gastric cancer patients. Acceptable LN removal and examination can contribute to accurate LN staging by affecting the clearance of possible metastatic nodes and stage transition^7^. Previous studies have revealed controversial findings regarding the correlation between the number of examined LNs and long-term survival. Some studies^8^ have shown that a higher number of removed LNs is associated with better survival, even for N0 disease, while other studies have found no such association^9,10^. Different biological behaviors of various tumor characteristics may affect the prognostic importance of LN examination. Most guidelines^5,6^ recommend examining at least 15 or 16 LNs, while the French Intergroup Clinical Practice Guidelines^11^ suggest ≥25 LNs. Additionally, a study has shown that survival advantages are achieved when the number of removed LNs is in the range of 25–29^12^. Li et al. have even suggested classifying node-negative patients as N1 stage when the number of removed LNs is insufficient^13^. Removing more LNs can cooperate with a more thorough clearance of potential tumor remnants, which may reduce recurrence and metastasis development, thus increasing long-term survival. It also helps prevent stage migration in these patients by avoiding staging a node-positive patient as negative, thereby preventing inappropriate treatment^7^.

Smith et al.^14^ found that for pT1/2N0 patients, each additional 10 LNs removed might result in a 7.6% increase in overall survival (OS). The question of how many LNs should be removed has been revisited in recent years, with different studies proposing varying numbers, and no consensus on a specific number has been reached^15,16^.

This study aimed to determine the relationship between the number of removed LNs and survival in patients who underwent radical gastrectomy and D2 LN dissection due to gastric cancer. Additionally, other factors affecting survival were examined.

## METHODS

The study group consisted of all cases who underwent gastrectomy and D2 LN dissection by a single team at Izmir Bozyaka Training and Research Hospital between 2013 and 2022. Prior to the launch of the study, ethical approval was obtained from the Ethics Committee of Non-invasive Ethics Committee of Izmir Bozyaka Training and Research Hospital on January 8, 2025, with decision number 2025-5. This study was conducted retrospectively based on data from a prospectively maintained database (retrospective cohort). Informed consent forms were obtained from the patients. Inclusion criteria were adenocarcinoma cases with stages I–III according to tumor node metastasis (TNM) staging. Cases with squamous cell carcinoma, gastrointestinal stromal tumors, sarcomas, neuroendocrine/carcinoid tumors, another cancer simultaneously or previously, and recurrent gastric cancer were excluded from the study.

The dependent variables of the study were 1-year and 3-year survival rates. Independent variables included the number of removed LNs, age, gender, tumor stage, operation time, presence of comorbidities, tumor status at the surgical margin, lymphovascular invasion, and perineural invasion. The diagnostic accuracy of the number of removed LNs and age in predicting survival was analyzed using receiver operating characteristic (ROC) curves and the area under the curve (AUC) with a 95%CI. These variables were included in the Cox regression analysis model according to the cutoff points obtained from this analysis. Separate Cox regression models for 1-year and 3-year survival rates were created to calculate hazard ratios (HRs) and present them with CIs. The correlation between the number of removed LNs and survival was assessed using Pearson's correlation analysis. Statistical analysis was performed using SPSS version 25.0, with p<0.05 considered statistically significant.

## RESULTS

In this study, 68.3% of the cases were male, with a mean age of 68.3±12.8 years (34–88) and a median age of 65.0 years. The average number of removed LNs was 39.6±6.4 (7–96), with a median of 40, while the median number of pathological LNs was 6 (0–57). The average operation time was 299.2±78.1 (165–630) min, with a median of 300 min. The average follow-up period for 1-year survival was 10.2±3.3 (2–12) months, with a median of 12 months, while for 3-year survival, it was 22.5±13.0 (2–36) months and 24 months, respectively. The 1-year survival rate was 72.3%, and the 3-year survival rate was 40.4%. Descriptive characteristics are presented in Table 1.

**Table 1:** Descriptive characteristics of the cases.

Feature		n (%)
Gender (n=101)		
	Female	32 (31.7)
	Male	69 (68.3)
Age group (n=101)		
	Under 70 years	64 (63.4)
	70 years and older	37 (36.6)
Stage (n=101)		
	1	12 (11.9)
	2	22 (21.8)
	3	67 (66.3)
Comorbidity (n=101)		
	Present	27 (26.7)
	Absent	74 (73.3)
Number of removed LNs (n=99)		
	Less than 35	38 (38.4)
	35 or more	61 (61.6)
Surgical margin (n=101)		
	Negative	92 (91.1)
	Positive	9 (8.9)
Vascular invasion (n=90)		
	Present	38 (42.2)
	Absent	52 (57.8)
Perineural invasion (n=90)		
	Present	49 (54.4)
	Absent	41 (45.6)
1-year survival		
	Dead	28 (27.7)
	Alive	73 (72.3)
3-year survival		
	Dead	56 (59.6)
	Alive	38 (40.4)

LN: lymph node.

When examining factors associated with 1-year survival, having fewer than 35 removed LNs significantly increased the mortality risk by 3.25 times (p=0.011, HR 3.25, 95%CI 1.31–8.11). Additionally, being 70 years or older (p=0.005, HR 3.74, 95%CI 1.50–9.33) and having lymphovascular invasion (p=0.013, HR 4.63, 95%CI 1.38–15.50) significantly increased the risk of death (Table 2).

**Table 2:** Factors affecting 1-year and 3-year survival: Cox regression analysis results.

Characteristic (reference group)		One-year survival (Ex)		Three-year survival (Ex)	
		p	HR (95% CI)	p	HR (95% CI)
Number of lymph nodes removed (≥35)	<**35**	**0.011**	3.25 (1.31–8.11)	0.096	1.69 (0.91–3.15)
Gender (female)	**Male**	0.674	0.81 (0.31–2.14)	0.240	0.67 (0.35–1.30)
Age group (<70 years)	≥**70**	**0.005**	3.74 (1.50–9.33)	**<0.001**	4.11 (2.09–8.09)
Comorbidities (none)	**Present**	0.897	1.06 (0.44–2.58)	0.732	0.89 (0.47–1.69)
Surgical margin (negative)	**Positive**	0.106	3.10 (0.79–12.24)	**0.038**	2.85 (1.06–7.66)
Lymphovascular invasion (none)	**Present**	**0.013**	4.63 (1.38–15.50)	0.203	1.61 (0.77–3.33)
Perineural invasion (none)	**Present**	0.122	0.35 (0.09–1.33)	0.456	1.36 (0.61–3.02)
Stage 1	**Stage 2**	0.263	0.38 (0.07–2.09)	0.872	0.91 (0.28–2.99)
	**Stage 3**	0.992	1.01 (0.22–4.72)	0.101	2.54 (0.83–7.76)
Duration of surgery		0.912	1.00 (0.99–1.01)	0.170	1.00 (0.99–1.01)

Statistically significant values are denoted in bold. CI: confidence interval; Ex: expected survival; HR: hazard ratio.

For 3-year survival, no significant relationship was found between the number of removed LNs and mortality risk (p=0.096, HR 1.69, 95%CI 0.91–3.15). However, being 70 years or older (p<0.01, HR 4.11, 95%CI 2.09–8.09) and having tumor presence at the surgical margin (p=0.038, HR 2.85, 95%CI 1.06–7.66) significantly increased the risk of death (Table 2). A positive correlation was found between the number of removed LNs and survival, with a moderate, significant positive correlation for 1-year survival (r=0.28, p=0.006) and a weak, significant positive correlation for 3-year survival (r=0.24, p=0.018) (Table 3).

**Table 3:** Correlation between number of removed lymph nodes and survival.

		One-year follow-up	Number of lymph nodes removed
1-year follow-up	Pearson's correlation	1	0.275*
	Sig. (two-tailed)		0.006
	n	101	99
3-year follow-up	Pearson's correlation	1	0.238
	Sig. (two-tailed)		0.018**
	n	101	99

* Correlation is significant at the 0.05 level (2-tailed).

** Correlation is significant at the 0.01 level (2-tailed).

## DISCUSSION

Our study thoroughly examined 1-year and 3-year survival rates and demonstrated that as the number of removed LNs increased, both 1-year and 3-year survival rates improved. Additionally, multivariate analysis showed that removing 35 or more LNs increased survival rates by 3.2 times for 1 year and 1.6 times for 3 years. However, this was only statistically significant for the 1-year survival rate. Furthermore, our study found that both the presence of a tumor at the surgical margin and advanced age were associated with decreased survival rates.

In a joint study conducted between 2010 and 2016 in the USA and China, which included 7,228 patients from the USA and 1,468 from China, it was shown that as the number of removed LNs increased, the number of pathological LNs also increased. In our study, the number of removed pathological LNs was 6. Survival correlation analysis revealed that both 1-year and 3-year survival rates improved as the number of removed LNs increased. This study showed that removing 33 or more LNs increased survival in both China and the USA. Additionally, in this study, removing 33 or more LNs improved survival in both node-positive and node-negative cases in the USA patient group, while in the Chinese patient group, the number of removed LNs increased survival in the node-positive group but did not significantly improve survival in the node-negative group^7^. Zhao et al., in another study of 2,246 cases, indicated that 15 LNs were insufficient, especially for patients with T2–4 or N1–3, and recommended removing at least 25 LNs^12^. Liu et al. confirmed that removing more than 25 LNs consistently showed the best OS for stages I–III^17^. Chen et al. found that at least 25 LNs could represent a superior criterion for N2–N3 stage patients^18^. Wang et al. also found that the contribution of removed LNs varied according to the T stage of the disease and could range between 26 and 45^4^. Similarly, Zhao et al.^3^ recommend the removal of at least 24 LNs in T2–4 disease. Another recent article suggested that for accurate staging in signet-ring cell gastric cancer, at least 22 LNs should be removed, and for a survival advantage, 44–52 LNs should be removed^15^. Li et al. ^19^ found that even in N0 patients who received neoadjuvant chemotherapy, survival improved when the number of LNs removed was at least 24. In our series, an average of 39 LNs was removed.

Reviewing these studies, it is evident that the number of removed LNs still varies significantly. We believe that a more standardized D2 dissection and additional effort are needed to achieve a specific range of these numbers. However, there is concern that increasing the number of removed LNs could lead to more complications and the uncertainty of whether it would contribute to survival. Nevertheless, D2 lymphadenectomy can be beneficial if postoperative events are avoided^20^. In our study, excluding patients who survived less than 3 months, mortality risks decreased up to 33 LNs. Encouragingly, surgical experience and quality have been improving with the reduction of postoperative events^21^.

Wang et al.'s study examined the removed LN numbers according to tumor T stage. They found that the removed LN numbers was an independent risk factor associated with the prognosis of patients with pT1–pT4 stage gastric cancer. The risk of mortality in patients increased with the number of removed LNs but was not constant. Increasing the number of LNs extended long-term survival in patients with pT1, pT2, and pT4 stages but did not improve long-term survival in patients with pT3 stage cancer. For pT1 stage patients, at least 26 LNs are recommended to be removed. For pT2 stage patients, at least 31 LNs are recommended. For pT4 stage patients, 45 LNs are recommended^4^. Hu et al.^22^ recommended the removal of at least 32 LNs in the presence of N3.

Our limitations include the relatively small number of cases, working with a heterogeneous group due to patients being at different stages, a single-center experience, and a short follow-up duration.

## CONCLUSION

In gastric cancer, survival can be improved with extensive surgery. Based on recent developments and our results, increasing the number of LNs removed through more extensive dissection can improve survival. Therefore, LN dissection should be performed as maintained by guidelines with increased effort to remove a higher number of LNs.

## Data Availability

The datasets generated and/or analyzed during the current study are available from the corresponding author upon reasonable request.
